# 3D bioprinting of complex tissues in vitro: state-of-the-art and future perspectives

**DOI:** 10.1007/s00204-021-03212-y

**Published:** 2022-01-10

**Authors:** Yi Xiang, Kathleen Miller, Jiaao Guan, Wisarut Kiratitanaporn, Min Tang, Shaochen Chen

**Affiliations:** 1 Department of NanoEngineering, University of California San Diego, La Jolla, USA; 2 Department of Electrical and Computer Engineering, University of California San Diego, La Jolla, USA

**Keywords:** 3D printing, Tissue model, In vitro model, Toxicity screening, Tissue engineering, Biomaterials

## Abstract

The pharmacology and toxicology of a broad variety of therapies and chemicals have significantly improved with the aid of the increasing in vitro models of complex human tissues. Offering versatile and precise control over the cell population, extracellular matrix (ECM) deposition, dynamic microenvironment, and sophisticated microarchitecture, which is desired for the in vitro modeling of complex tissues, 3D bio-printing is a rapidly growing technology to be employed in the field. In this review, we will discuss the recent advancement of printing techniques and bio-ink sources, which have been spurred on by the increasing demand for modeling tactics and have facilitated the development of the refined tissue models as well as the modeling strategies, followed by a state-of-the-art update on the specialized work on cancer, heart, muscle and liver. In the end, the toxicological modeling strategies, substantial challenges, and future perspectives for 3D printed tissue models were explored.

## Introduction

In vitro tissue models have greatly advanced our understanding of the pharmacological and toxicological processes of a wide range of treatments and chemicals (Davila et al. 1998). Such models are efficient, low-cost, and non-cruel recapitulations of native tissues, and their development has sped the discovery of various medications (Madorran et al. 2020), as well as the development of environmental pollution prevention and labor protection approaches. In vitro tissue models have evolved from simple two-dimensional (2D) monocultures into more advanced three-dimensional (3D) structures, such as organoids, dynamic culture systems, micro-tissues, organ-on-chip devices, and other combinations (Braun et al. 2021; Duval et al. 2017). Accurate recapitulation of native physiology, such as cell composition, biophysical and biochemical signaling, as well as microarchitecture, could result in greater substantive response when drawing correlations between in vitro and in vivo conditions (Lelièvre et al. 2017).

3D bioprinting has emerged as an intriguing approach for the production of complex in vitro models, by which means cells and/or their supporting scaffold are precisely deposited, localized, or joined in user-defined geometries and dimensions. With an ever-expanding range of available biomaterials (Yu et al. 2020a, b) and biocompatible processes (Ashammakhi et al. 2019), 3D bioprinting has aided in the tailored control over microarchitecture, extracellular matrix (ECM) construction, and cell deposition for the establishment of in vitro models, particularly the recapitulation of complex tissues (Ma et al. 2018a, b), and has resulted in significant accomplishments in moving the field forward in recent years.

Here, we present a state-of-the-art review on the in vitro complex tissue model constructions based on 3D bioprinting. We begin with an overview of 3D printing techniques, biomaterials and their use in in vitro tissue construction, and then move on to discussing pioneering work in cancer, heart, liver, and muscle in vitro models for biological studies, drug screening, and toxicity investigations. In the concluding section, we also explore the applications, challenges, and future perspectives of 3D bioprinting technologies and tissue modeling.

## 3D bioprinting technologies and biomaterials

### 3D bioprinting technology

3D bioprinting refers to a type of additive manufacturing, specifically a layer-by-layer fabrication technique that was originally born out of a need for rapid prototyping and has since enjoyed advancement into a fast, customizable fabrication method across many fields. 3D bioprinting technology allows for flexibility in both material choice and design paradigm—in the context of tissue engineering, the ability to incorporate biomaterials and cells inherently allows for 3D bioprinting. As 3D bioprinting becomes more ubiquitous, more research into bioprinting techniques has emerged, allowing for the fabrication of a wide range of biocompatible constructs, and cell-encapsulated tissues, and organ models.

#### Inkjet-based

**Fig. 1:**
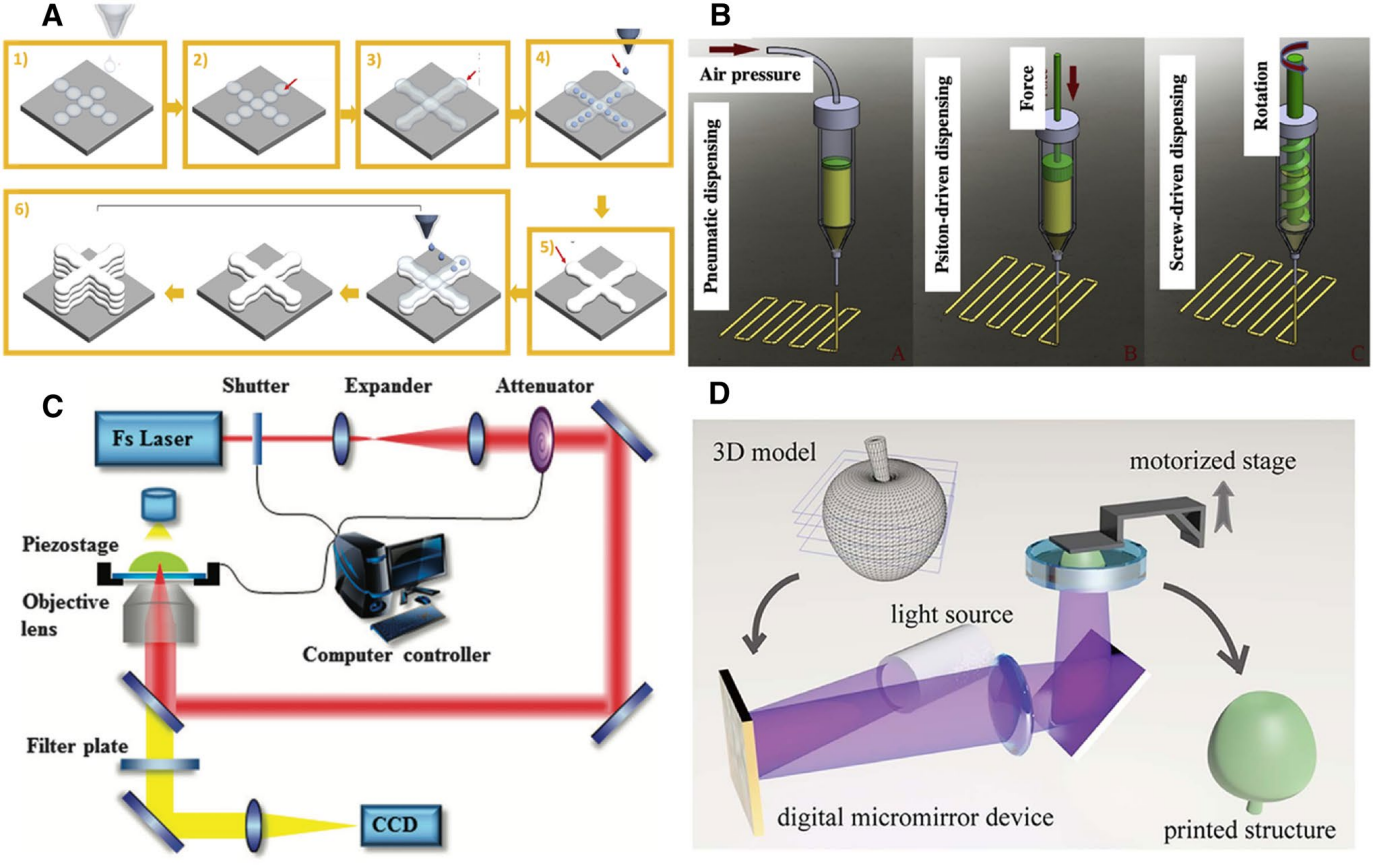
Schematic illustrations of 3D bioprinting processes. **A** Inkjet-based (Gudapati et al. 2016): (1) hydrogel precursor droplet deposition on a substrate; (2) droplet spreading; (3) droplets assembling into lines; (4) cross-linker droplet deposition; (5) hydrogel crosslinking; (6) process repeated for layer-by-layer fabrication; **B** extrusion-based (Derakhshanfar et al. 2018); **C** TPP-based (Xing et al. 2015); **D** DLP-based (You et al. 2020)

A typical inkjet-based bio-printer is shown in Fig. 1A (Patel 2016). It dispenses droplets of low-viscosity bio-ink from a ‘printhead’ containing arrays of small nozzle apertures to form patterns and then stabilizes the structure by photo-crosslinking or thermal gelation (Yu et al. 2020a, b). Typical inkjet printer designs include: a bioink storage chamber(s), actuators to both guide bioink(s) to the nozzle and form the droplets, and stage/control systems for three-axial movement. There are three main categories of inkjet bioprinting methods: continuous-inkjet, droplet-on-demand, and electro-hydrodynamic jet bioprinting, all of which differ in their method of bioink droplet deployment control. The continuous-inkjet method extrudes streams of bioink droplets, which lack precise droplet control. The droplet-on-demand bioprinter improves droplet control by creating individual droplets at required times by pressurizing the bioink storage chamber, using thermal, piezoelectric, or electrostatic-based actuators. The electro-hydrodynamic jet bioprinter generates droplets by pulling the bio-ink through the nozzle instead of a pushing method with droplet-on-demand bioprinters (Derakhshanfar et al. 2018; Gudapati et al. 2016).

In general, inkjet-based 3D bioprinting has the benefit of precise material deposition with a reasonable printing speed. The utilization of bioink droplets results in minimal material waste and low cost, with a high cell viability (except in thermal inkjet bioprinting). The bioink chamber and nozzle design also allow efficient material replacement and multi-material printing. However, a drawback to inkjet printing is that small nozzle apertures place a lower limit on cell densities in the bioinks—higher cell densities can cause nozzle clogging. Additionally, the low viscosity required for printing may result in limited mechanical strength in the final printed structure. Furthermore, the droplet form of the material causes a limited printing resolution (Gudapati et al. 2016; Mobaraki et al. 2020).

#### Extrusion-based

Extrusion-based bioprinting is another widely used bioprinting technology (Fig. 1B). Similar to inkjet printing, extrusion-based printing also extrudes material from a nozzle printhead, but instead of dispensing individual droplets, a typical extrusion printer extrudes a continuous flow of viscous bioink filament. The bioink viscosity used in extrusion bioprinting ranges from 30 to 60 kPa s (Derakhshanfar et al. 2018; Mobaraki et al. 2020). Higher-viscosity bioinks allow for the use of correspondingly higher viscous biomaterials and higher-density cell encapsulation, both of which are beneficial for tissue and organ fabrication. Extrusion-based bioprinter resolutions are limited by the size of the nozzle aperture and material characteristics, and also experience slower printing speeds due to the scanning nature of its printing movement. For both the extrusion-based and inkjet-based bioprinting, supporting structures are needed if creating overhanging 3D structures, which may introduce longer printing times as well as material/cell wastage. The overall fabrication time will vary based on the complexity of the 3D structure (Yu et al. 2020a, b).

#### Laser polymerization-based

Laser-based bioprinting methods feature the use of a precisely controlled, focused laser beam to effect patterned photopolymerization in a prepolymer solution. Among them,  two-photon polymerization (TPP) bioprinting uses femtosecond laser pulses to achieve submicron level precise printing (Fig. 1C) (Claeyssens et al. 2009; Zhang and Chen 2011; Xing et al. 2015). The high precision afforded by TPP printing allows for the production of micro- and nano-scale tissue scaffolds and vasculatures. However, to ensure precise synchronization between the motion and laser pulses, the TPP printing speed is often limited, and thus requires long fabrication times for complex and/or large structures (Yu et al. 2020a, b). After printing, the un-polymerized residual solution will need to be removed to reveal the printed structure, which causes material waste; however, this material may function as a soft, supportive structure during the actual printing process, thus eliminates the need for designated supporting structures (Yu et al. 2020a, b). TPP printing allows for a variety of material viscosities, which is beneficial for the varied conditions of tissue and organ printing, but higher laser powers can cause thermal damage to cells, resulting in reduced cell viability (Derakhshanfar et al. 2018; Hopp 2012).

#### Digital light processing (DLP)-based

DLP-based bioprinting is an emerging photopolymerization-based bioprinting technique that addresses the primary limitations of previous bioprinting methods—speed and spatial resolution for tissue and organ fabrication. Compared to other methods that require point-by-point or line-by-line scanning to create an individual layer, DLP-based bioprinting drastically reduces the printing time by enabling the projection of an entire 2D design plane at once. A DLP printer’s core piece of hardware is a digital micro-mirror array device (DMD), a programmable micro-opto-electromechanical chip with an array of micro-mirrors—this can be used to spatially pattern an incoming light source, such as a 365 nm ultraviolet or 405 nm visible light, to photo-polymerize a vat of prepolymer solution (Fig. 1D). The XY resolution of the printed structure is defined by the projection from an individual micro-mirror on the DMD chip, which may be as low as 3–5 µm (and can vary based on intermediate optics and prepolymer characteristics) (Lu et al. 2006), with a typical layer-by-layer Z-resolution limited by the material refill process between subsequent layers (Yu et al. 2020a, b). The Chen group later developed a dynamic optical projection stereolithography (DOPsL) to continuously move the *Z*-axis during printing, resulting in smooth side walls for printed structures (Zhang et al. 2012), and applied this for pre-vascularized tissue printing (Zhu et al. 2017). Further additions to printing efficiency have been researched as well, to go beyond layer-by-layer into volumetric additive manufacturing, where the entire volume is printed at once. One approach is the holographic volumetric 3D fabrication system, which uses three orthogonal light beams with phase-only patterns to print the entire 3D structure in a single step (Shusteff et al. 2017). Another example is the computed axial lithography (CAL) technology, which utilized a DLP projector to illuminate their prepolymer vat from a single side, axially rotating the vat while modulating the projected light’s phase pattern (Kelly et al. 2019). Such volumetric additive manufacturing fabrication techniques are not limited in printing time by the *Z*-axis, but spatial resolution can suffer due to limitations in holographic precision. Recent efforts by Saha et al. (2019) to address these spatial resolution limitations utilize femtosecond projection two-photon lithography (FP-TPL), which combines the high precision afforded by TPP with the high-throughput nature of DLP-based printing, allowing simultaneous spatial and temporal focusing.

Overall, DLP-based bioprinting enables rapid micro-scale bio-fabrication by plane-wise or even volume-wise printing with the use of a DMD. The high-throughput nature of the process is greatly beneficial for the fabrication of tissues and organs, taking into consideration the time-sensitive nature of live cells and tissues. Additionally, the micro-scale precision afforded by DLP-based printing is similarly beneficial for producing the fine features of native physiology, such as multi-tissue compartments and microvasculature. The relatively low power of the light sources and exposure times typically used also ensure cell viability (Ruskowitz and Deforest 2019). One drawback to DLP-based bioprinting is its pre-filling of a vet of bioink which if not used and recycled, would go to biological waste.

### Biomaterials in 3D printing

Biomaterials form the basis of the matrix or substrate of the final printed structure, and are critical in the in vitro modeling of complex tissues—they provide crucial physical and chemical signals, and can have significant impacts on cell activities, such as adhesion, metabolism, proliferation, differentiation, and migration. For example, the stiffness of the extracellular matrix as a biophysical cue has been found to be deterministic in the viability, differentiation, and migration of a variety of cell types (Gasiorowski et al. 2013). Material porosity crucially aids in the transfer of substances, cell proliferation, and migration (Li et al. 2021). The texture of the substrate surface, such as bio-macromolecule alignment and micro/nanostructure, influences adhesion, migration, and maturation, having a substantial impact on tissue organization, remodeling, and development (Fleszar et al. 2018; Liu et al. 2020). Meanwhile, biochemical stimuli are conveyed via the material's backbone or network. The presence of cell adhesion motifs, such as RGD and GFOGER, for example, is crucial for the modeling with adherent cells. Bioactive small molecules, such as metal ions (Yang et al. 2021) and protein imitating peptides (Liu et al. 2010), can be integrated into the material to trigger specific biochemical signaling pathways for specific purposes. Therefore, in addition to taking into account the printing technique, the biomaterials used for in vitro tissue modeling via 3D printing must be carefully chosen based on the tissue of interest, as well as the study's purpose and scientific inquiry. Here, we review the most commonly used biomaterials for 3D bioprinting, with a focus on their characteristics and applications.

#### Gelatin

From a chemistry perspective, gelatin is a polypeptide, generated from the hydrolysis of collagen. Gelatin has become one of the most widely used materials in tissue engineering, due to the abundance of cell-adherent RGD motifs in its backbone, excellent biocompatibility, good biodegradability, and low immunogenicity. Its moderate translucency, viscoelasticity and strength also make it appropriate for various bio-fabrication and bioprinting methods. GelMA, a gelatin-based biomaterial in which the primary amines in the lysine backbone of gelatin are replaced with methacrylate groups to facilitate photo-initiated free-radical polymerization, is one of the most common (van Hoorick et al. 2019). For example, GelMA-based micro-constructs with encapsulated conjunctival stem cells were created using DLP-based bioprinting (Zhong et al. 2021a, b), where the GelMA provided a nurturing 3D environment that maintained stem cell phenotype and differentiation potency while maintaining the vitality. Click chemistry methods have been emerging as a way to modify gelatin because of its high efficiency, high selectivity, and minimal side reactions at mild reaction conditions. Gelatin, for example, was modified with norbornene and thiol to allow for photo-reactive thiol-ene crosslinking (Yu et al. 2020a, b). The highly selective reaction enabled high-order programmable bio-functionalization and tailored regulation of the mechanical properties of the hydrogel matrix. Click-crosslinked gelatin can also be obtained through the Diels–Alder reaction (García-Astrain et al. 2014) and the carbodiimide reaction (Cammarata et al. 2015).

#### Hyaluronic acid

Hyaluronic acid (HA) is a glycosaminoglycan composed of d-glucuronic acid and *N*-acetyl-d-glucosamine units. The abundance of reactive groups in its backbone allowed various crosslinking and chemical modification strategies to accommodate it for different printing methods. For example, hyaluronic acid glycidyl methacrylate (HAGM) has been synthesized for photo-assisted 3D printing (Liu et al. 2020). Furthermore, adamantane and beta-cyclodextrin were linked to HA to enable a non-covalent guest–host assembly, which introduces shear-thinning behavior into the hydrogel and aids the extrusion-based printing by adding a temporary mechanical support in the printed structure (Ouyang et al. 2016). In native physiology, HA is a key component of the ECM in cartilage, the eyeball, the brain, and a variety of other tissues, as well as a participant in tissue microenvironment, cell signaling, and tumor progression. Thus, in certain cases, HA is required for in vitro modeling of complicated tissue. To capture the HA-rich ECM in cartilage tissue, for example, methacrylated HA was introduced to the bioink as a matrix for in vitro chondrogenesis, resulting in enhanced tissue organization (Mouser et al. 2020). In another example, HAGM was used to print the scaffold to support the quiescence state of the limbal stem/progenitor cells (LSC), while the cells remained active when encapsulated in GelMA. The distinct states of the cells in different biomaterials enabled the fabrication of dual-state cells in a single construct, addressing a better mimicry of the native LSC niche (Zhong et al. 2021a, b). For further applications, HA can be modified for click chemistry crosslinking via a variety of mechanisms.

#### De-cellularized extracellular matrix (dECM)

De-cellularized extracellular matrix (dECM) of the tissue of interest can be manufactured and applied for 3D printing for enhanced recapitulation of native physiological microenvironments. dECM is not only made up of biopolymers like collagen, fibrin, and glycosaminoglycans as a supporting framework, but also retains the host tissue’s native biochemical signaling molecules. While dECM’s thermal gelling nature makes it suitable for extrusion-based bioprinting, its poor mechanical strength makes it difficult to use as a bio-scaffold on its own—as a result, substantial efforts have been made to composite dECM with other materials. For DLP printing of an in vitro liver lobule tissue, liver dECM was produced and combined 1:1 with GelMA. With the complex biochemical cues given, a stable physiological-mimicking environment for HepG2 3D culture was achieved (Ma et al. 2018a, b).

#### Alginate

Alginate is a brown algae-derived copolymer comprising beta-d-mannuronate and alpha-l-guluronate. Alginate is a popular choice for complex in vitro tissue modeling due to its simultaneous nature of high biocompatibility and bio-inertness. As a result, alginate makes a good choice for investigations that require minimal degradation as well as precise control of scaffold/substrate stiffness. Alginate possesses the appropriate rheological properties and ion cross-linking capabilities for inkjet and extrusion printing, and can also be chemically modified using 2-aminoethyl methacrylate hydrochloride (AEMA) for photo-assisted printing methods for a broader applicability. Alginate can also build an interpenetrating polymer network (IPN) with other hydrogel materials like collagen and dextran to provide even more customized stiffness and multiplexed bioactivity. For example, alginate was incorporated with collagen to generate the IPN with a tunable storage modulus of 49–419 Pa, the variation of which triggered a reversible state of cancer-associated fibroblast between inflammatory state and myofibroblastic state (Cao et al. 2021).

#### Synthetic polymers

Synthetic polymers, in contrast to biopolymers formed from natural sources, are of tremendous interest due to the inherent consistency and feature tailorability that come with scalable industrial manufacturing. One example is polyethylene glycol (PEG), a synthetic polymer extensively used in tissue engineering applications due to its biocompatibility, non-immunogenic degradation and high capacity for modification. Many functional groups, such as protein-mimicking peptides, growth factors, signaling molecules, and significantly, acrylate groups for photo-polymerization, can be added due to the abundance of reactive hydroxyl groups in the backbone. The mechanical properties of a PEG-based hydrogel, on the other hand, are determined by its molecular weight, the concentration and density of crosslinking synergistically, implying that the substrate's stiffness can be customized by demand. The Young’s modulus of PEG diacrylate (PEGDA) ranges from 668 Pa to 2 kPa as the molecular weight varies from 575 to 20,000 (Vannozzi et al. 2018). Polymers made from various monomers could be used for specific requirements in the substrate or scaffold for in vitro complicated tissue modeling. For example, poly (glycerol-co-sebacate), polyurethane, or polycaprolactone can be used to form an elastic substrate, collectively providing a varied range of elastic strength and degradation profile.

## State-of-the-art of in vitro tissue models

Along with the evolution of 3D bioprinting techniques and biomaterials, in vitro models of a variety of tissues, organs and diseases have been developed, evaluated, optimized and brought into use. Here, taking cancer, heart, liver and muscle as examples, we review the pioneering and representative work in the field.

### Cancer

Cancer remains a significant public health issue worldwide due to its high occurrence, mortality, and economic impact. 3D bioprinting offers an opportunity to advance the in vitro modeling of various cancers types, such as brain cancer (Tang et al. 2020, 2021), pancreatic cancer (Hakobyan et al. 2020), liver cancer (Ma et al. 2018a, b), lung cancer, colorectal cancer, ovarian cancer (Xu et al. 2011), breast cancer (Hribar et al. 2015; Zhu et al. 2016), and metastatic models (Meng et al. 2019; Zhu et al. 2016), owing to its ability to recapitulate the complex cellular and material heterogeneities.

#### Brain cancer

**Fig. 2:**
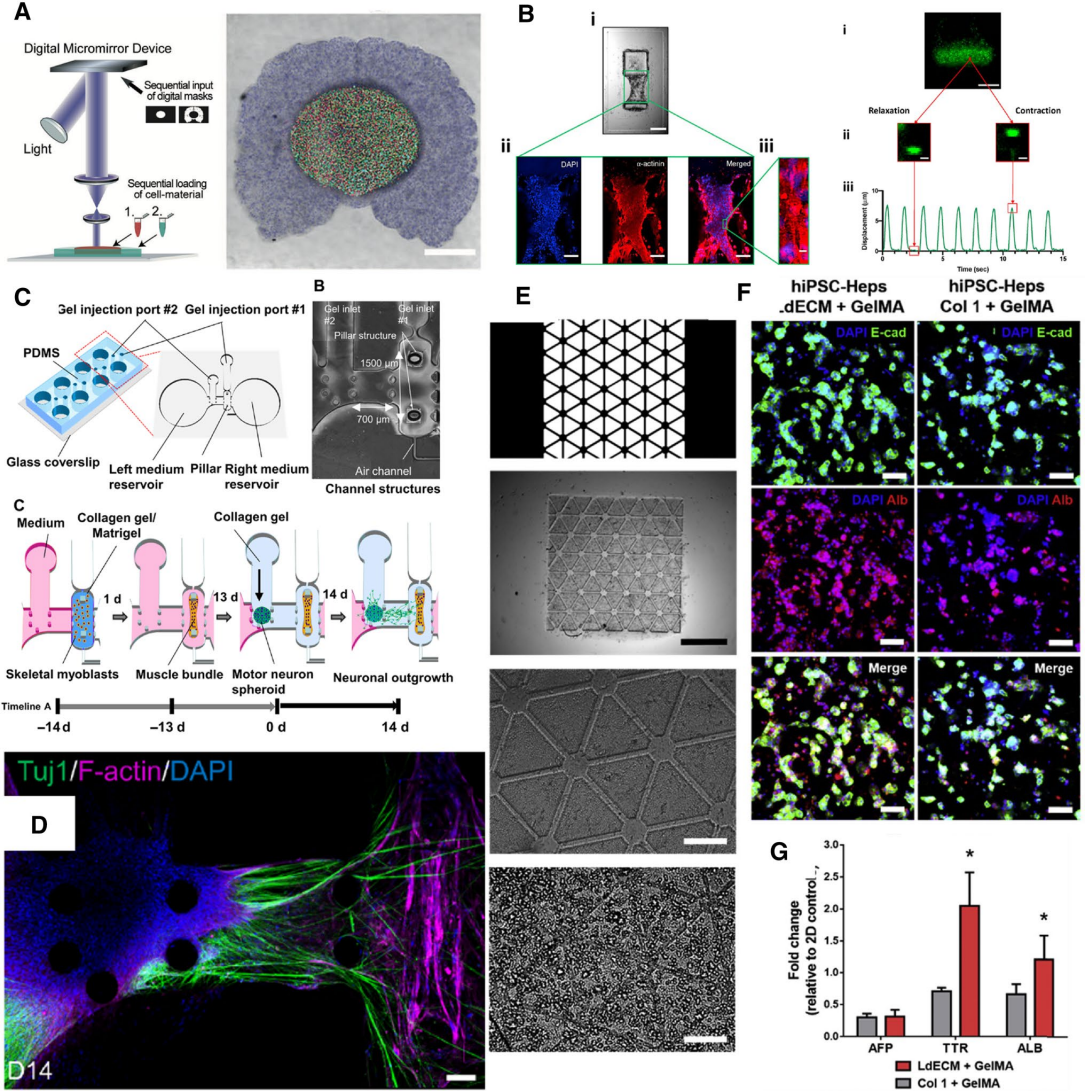
Examples of recently developed 3D in vitro models. **A** Fabrication schematic and example print of a GBM model (Tang et al. 2020); **B** Remodeling, maturation and contraction characterization of a pillar-based heart micro-tissue (Miller et al. 2021) for high-throughput screening; **C** Illustration of the construction of an ALS model, and **D** ALS patient-derived motor unit remodeled in the ALS model (Osaki et al. 2018); **E** High-resolution printing of patient-derived liver model in a ECM-mimetic bioink, and **F**, **G** better remodeling of the phenotype and gene expression profile of iPSC-derived hepatocytes in the ECM-mimetic microenvironment (Yu et al. 2019)

Tang et al. developed multicellular glioblastoma (GBM) models using DLP-based bioprinting (Tang et al. 2020). Patient-derived GBM stem cells (GSCs), macrophages, neural progenitor cells, and astrocytes were fabricated into a defined spatial organization to form a brain tumor within brain architecture, recapitulating immune interactions and functional dependencies in 3D microenvironment (Fig. 2A). Tumor cells demonstrated higher drug resistance and invasion capacity with the inclusion of macrophages. Extrusion-based bioprinting was also used to generate GBM models composed of GSCs, patient-derived GBM-associated stromal cells, and microglia in an alginate-based hydrogel (Hermida et al. 2020). Alginate hydrogels were modified with RGDs for better cell attachment. The 3D GBM models demonstrated enhanced resistance to cisplatin which failed in many clinical trials but showed promising efficacy in 2D cell cultures. 3D models have a potential application for more reliable efficacy evaluations.

#### Pancreatic cancer

Hakobyan et al. used laser-assisted bioprinting (LAB) for generation of pancreatic ductal adenocarcinoma (PDAC) spheroid arrays consisted of both acinar and ductal cells in GelMA (Hakobyan et al. 2020). These models were used for interrogation of different factors that contribute to the precursor PDAC lesions at the early stage PDAC onset and progression. Xu et al. (2019) developed PDAC models with biomimetic materials consisted of surface-engineered cellulose nanofibrils (CNFs) and photo-crosslinkable galactoglucomannan methacrylates (GGMMAs), and a UV-assisted extrusion-based printing techniques. The bioinks demonstrated promising biocompatibility and supported pancreatic cancer cell and dermal fibroblast proliferation. Utama et al. reported a drop-on-demand method to rapidly form PDAC models in 96-well format (Utama et al. 2021). Tunable biological and mechanical properties were enabled by the 4-arm PEG-based polymers that can form hydrogels within seconds.

#### Lung cancer

Mondal et al. utilized extrusion-based printing and a sodium alginate–gelatin hydrogel to develop non-small cell lung cancer (NSCLC) co-culture models with patient-derived xenograft cells and cancer-associated fibroblasts (Mondal et al. 2019). The methods demonstrated high printability and good cell viability. After two weeks of in vitro culture, the NSCLC spheroids could reach a diameter ranging from 50 to 1,100 µm, creating hypoxic cores within the spheroids for further research. Cellular crosstalk created by the co-culture system was confirmed by upregulation of specific genes, such as vimentin and α-SMA. Wang et al. reported a method that combined low-temperature molding and 3D bioprinting technique to fabricate a lung cancer model (Wang et al. 2018). A biomimetic 3D hydrogel grid scaffold was generated with gelatin, sodium alginate, and lung cancer cells A549/95-D. Cell proliferation plateaued after two weeks of culture and had a viability over 90%.

#### Colorectal cancer

Chen et al. reported a 3D printed colorectal cancer (CRC) model that closely mimicked the physiological functions and cellular crosstalk between the tumor cells and tumor-associated stromal cells (Chen et al. 2020). Co-culture of colorectal cancer cells, cancer-associated fibroblasts, and tumor-associated endothelial cells on bio-printed scaffolds reprogrammed normal stromal cells into tumor-associated phenotypes. Cellular processes and vascularization were observed, and could help elucidate oncogenesis factors and evaluate the efficiency of potential drugs.

Tariq et al. utilized 3D magnetic bioprinted CRC models to investigate P-glycoprotein associated multidrug resistance (MDR) in cancer treatment (Tariq et al. 2020). The authors delivered siRNA, designed against MDR1 gene to silence the gene in Caco-2 cells and studied the role of MDR-1 gene in both 2D and 3D culture conditions. The 3D model was compared to 2D culture and demonstrated that the knockdown of MDR1 gene in colorectal carcinoma cells can significantly reduce the tumor cell migration in both 2D cell culture and 3D bioprinted models.

#### Breast cancer

Langer et al. (2019) reported generation of a multicellular scaffold-free tumor tissue representing subtypes of breast cancer and pancreatic cancer using 3D printing. The multiple cell types within the printed structure could self-organize into biomimetic morphologies and secreted their own ECMs to reform the tissues. Incorporation of patient-derived cells into the models offeres a translational tool for investigating the therapeutic responses, potential oncogenic endpoints, and crosstalk between different cell types relevant to individual patients.

Vinson et al. (2017) investigated epithelial-adipose interactions in breast cancer using a 3D-printed breast cancer model. Patient-derived breast cancer cells MCF-7 and MDA-MB-231 and differentiated adipocytes were spatially patterned by laser direct-write bioprinting technique. Investigations of early onset of cancer cell invasion through cellular and tissue-level interactions in the adipose tissue were enabled by the 3D models.

Duchamp et al. (2019) established a sacrificial bioprinting strategy to generate biomimetic mammary duct cancer models to study the oncogenesis and invasion processes of breast cancer. The models were first generated with GelMA into duct-like structures, and the channels were then populated with MCF-7 cells, which was reported to have relatively low invasiveness. Agarose was used as a sacrificial material for convenient extraction. The breast cancer model could be cultured over 24 days and outward invasion of cancer cells into the duct-like matrix was observed. This proof-of-concept model demonstrates the potential value of 3D printed models in studying the mechanism of oncogenesis of breast cancer.

### Heart

According to a study by the American Heart Association (2021), cardiovascular disease (CVD) affects nearly 50% of the US population and accounted for more than 360 billion dollars in consumer costs from 2016 to 2017, thus making CVD of significant medical, scientific, and economic importance. To help delve into and improve research surrounding CVD, significant effort has been invested into state-of-the-art solutions, from 3D-printed cardiac patches for damaged hearts to engineered heart tissues (EHTs) for evaluating drug efficacy and toxicity (Dvir et al. 2011).

When evaluating a drug, researchers have commonly used traditional 2D cultures and/or animal models. Due to the lack of chemical and biophysical cues a 2D culture receives from its environment; specifically the cell–extracellular matrix, cell–cell, and tissue-level interactions; these cells are unable to properly recapitulate the response of a mature adult heart (Veldhuizen et al. 2019; Zuppinger 2019). On the other end of the spectrum, murine models are appealing as they can capture these environmental cues. However, inter-species differences in ion channels, biological pathways and pharmacokinetic properties negatively impact the predictive ability of these models for human hearts (Mathur et al. 2016). The need for a better predictive model of drug toxicity is paramount, as evidenced by the fact that 45% of post-approval drug withdrawal from the market is related to cardiovascular system issues (Ferri et al. 2013).

To address these issues, researchers have developed various cardiac models and EHTs. Using a variety of methods, researchers have formed tissues with measurable functionality (beating frequency and force) against drugs or toxins (Mathur et al. 2016; Nam et al. 2015; Veldhuizen et al. 2019). One such EHT is the flexible cardiac thin film, developed by the Parker group (Grosberg et al. 2011). The model was created by seeding cardiac cells onto a thin sheet of fibronectin-stamped polydimethylsiloxane (PDMS), with the stamped fibronectin encouraging an anisotropic cardiac orientation, which is important for optimal force generation. The contractility of the tissue was then evaluated by measuring the “curl” of the thin film that occurred during tissue contraction. After developing this initial model, the group then expanded on the original study with multiple derivations and improvements of the original model (Ahn et al. 2018; Lind et al. 2017; McCain et al. 2013; Wang et al. 2014). In one compelling study, the group investigated Barth syndrome, a cardiomyopathic disease caused by mutated TAZ (the gene Tafazzin), in Cas9-edited induced pluripotent stem cell cardiomyocytes (iPSC-CMs). Using the in vitro model to analyze sarcomere assembly, contractile stress generation, and ROS (reactive oxygen species) production differences in the modified iPSC-CM compared to the wild type, the researchers were able to successfully show that both the reintroduction of wild-type TAZ and that suppression of ROS by the mitochondria reversed cardiomyopathic symptoms (Wang et al. 2014).

However, many researchers have created much thicker tissues, as opposed to thin films, to better recapitulate a mature heart. One of the most common EHTs used in the field for thick tissues is a pillar design, where cardiac cells are attached to two anchor points, either pillars or wires (Hinson et al. 2015; Liu et al. 2020; Ma et al. 2019; Miller et al. 2021; Ronaldson-Bouchard et al. 2018; Tiburcy et al. 2020; Williams et al. 2021). These anchors are commonly formed from molded PDMS, which is then immersed in a suspension of cells and extracellular matrix (ECM), naturally forming a 3D tissue over multiple days of culture. Like the thin films, these models have also been specialized for disease types. For example, Hinson et al. investigated the effect of different titin protein mutations, an important protein for sarcomere functionality. Using the 3D pillar model, the researchers were able to examine the impact of different titin variants on tissue contractility (Hinson et al. 2015). In another study, Williams et al. developed an arrhythmic cardiac model by dosing culture models with methyl-beta cyclodextrin (MBCD). The MBCD induced arrhythmic behavior in the tissue, which subsequently lost calcium handling ability and exhibited fibrotic activity. This impacted the function of the tissue even after removal of MBCD from culture (Williams et al. 2021).

However, these thicker EHTs rely on passive tension for the cardiac cells to self-organize into an anisotropic orientation. To directly address this, some researchers have incorporated 3D bioprinting into the model by directly printing encapsulated cardiac cells into small lines stretching between two pillars (Fig. 2B) (Liu et al. 2020; Miller et al. 2021). This is significant, as alignment has been shown to increase the maturity of the iPSC-CMs, which thereby increases the ability of the cells to recapitulate the adult heart during drug and toxin testing (Guo and Pu 2020; Hirt et al. 2014). Moreover, since the cells are directly printed, the tissues can be aligned in a small spatial footprint, enabling high-throughput testing on a 96-well plate, as opposed to the 24- or 48-well format used by other pillar models (Miller et al. 2021).

Applications of 3D printing have also expanded into full heart and chamber models (Lee et al. 2019; Wang et al. 2021). However, the field still has a long way to go before regularly using these models for in vitro testing. In particular, the immaturity of iPSC-CMs, which are the preferred cardiomyocyte source, is a persistent issue (Hirt et al. 2014; Ronaldson-Bouchard et al. 2018). To fully repair or even recapitulate an adult heart, we need to have a cell source that is robust and mature. Nevertheless, 3D models have undoubtedly advanced the field of human cardiac research and will continue to do so as new techniques are developed.

### Liver

The liver functions natively as the primary site for metabolism and detoxification in the body, thus making it a popular tissue for in vitro modeling of screening drugs, mechanistic studies, and liver regeneration. A wide range of biomaterials, such as gelatin, alginate, GelMA, dECM, hyaluronan, and collagen in different combinations, as well as different cell sources, such as primary hepatocytes, liver cancer cell lines, and stem cell-derived hepatocytes, have been utilized for creating biomimetic structures of liver (Ma et al. 2016).

Kang et al. developed a vascularized liver lobule array model with hepatic cells and endothelial cells using preset extrusion bioprinting (Kang et al. 2020). Briefly, a preset cartridge with hepatic, endothelial, and lumen regions was fabricated and injected with bioink with hepatic cells, endothelial cells, and sacrificial materials, respectively. Spatially patterned models of different cell types demonstrated improved functional properties of liver, including higher albumin secretion and urea production, compared to mixed cell types with no spatial organization. Endothelial cells provided structural integrity of the model after culturing for a week. Grix et al. reported a perfusion-enabled liver model with twelve micro-channels open at both sides in a hexagonal structure (Grix et al. 2018). HepaRG cells and human stellate cells were patterned using stereolithography. Stable expression of tight junctions and metabolism markers was observed in the model. The channels within the bioprinted liver were shown to be perfusable.

Efforts have been made on developing more biomimetic bioink for liver tissue engineering. Several groups reported the benefits of using liver dECM for generating liver models, in terms of improved printability, mechanical properties, as well as biological properties (Kim et al. 2020; Lee et al. 2017; Yu et al. (2019) developed photo-crosslinkable liver-specific dECM bioinks to generate complex patient-specific liver models. HiPSC-derived hepatocytes were patterned into hexagonal microscale architectures and demonstrated high cell viability and improved maturation in dECM bioinks compared to collagen bioink (Fig. 2E–G). Mao et al. (2020) also reported using DLP-based bioprinting and GelMA/dECM to generate a micro-liver tissue with improved hepatic function restoration. Human-induced hepatocytes demonstrated improved viability and functionality in bioink with dECM compared to bioink with only GelMA. Mazzocchi et al. (2019) improved the printability of collagen I by mixing it with thiolated hyaluronic acid at various ratios. Primary hepatocytes and stellate cells were printed using the composite bioink which remained viable for two weeks. Gori et al. (2020) developed a thermo-responsive hydrogel with alginate and sacrificial Pluronic materials for culturing hepatic cells in 3D constructs. The Pluronic materials improved the diffusion properties of the hydrogel and supported better cell viability.

Goulart et al. (2020) compared using single cell dispersion, 2D HiPSC-hepatocytes, and HiPSC-hepatocyte spheroids for 3D printing with non-parenchymal cells. Single cell format had the worst performance, demonstrating reduced viability and hepatic functions after 18-day cultivation period. Loss of hepatic phenotype was also observed in single cell models. In contrast, spheroid-based models demonstrated improved functionality and stability.

Engineered human liver models have a steadily increased use in the pharmaceutical industry due to their improved functionality, maturation, and steady metabolism compared to 2D-cultured cells (Underhill and Khetani 2018). The drug acetaminophen demonstrated significantly increased sensitivity in 3D-printed liver models than their 2D counterparts (Gori et al. 2020). The liver models can also serve as tools for viral study and infectious viruses. Bioprinted liver models using optimized bioink consisting of alginate, gelatin, and dECM were efficiently transduced with adeno-associated virus (AAV) and supported adenovirus replication (Hiller et al. 2018).

### Muscle

Skeletal muscle is the most abundant tissue in the human body and is innervated by motor neurons through neuromuscular junctions (NMJs), crucial for both locomotion and the coordination of tasks through directional force generation via contraction and relaxation of myofibers (Grefte et al. 2007). Healthy skeletal muscle tissue possesses the ability to regenerate upon injury through the migration, proliferation, and differentiation of nearby satellite cells into mature functional myofibers (Greising et al. 2019), but can lose this regenerative capacity due to a number of factors, including but not limited to traumatic injury, aging, or diseases, such as amyotrophic lateral sclerosis (ALS), Duchenne muscular dystrophy (DMD), myotonic dystrophy (DM), spinal muscular atrophy, and myasthenia gravis (MG), which can lead to a reduction in quality of life (Larkindale et al. 2014, Cappello and Francolini 2017).

To understand the underlying mechanisms of muscular disorders to come up with novel therapeutic intervention strategies, research efforts have been made toward developing physiologically relevant in vitro skeletal muscle models to overcome the limitations imposed by current animal models, such as species–species pathological differences and response.

Microfluidic device serves as suitable candidates for developing in vitro NMJ platforms, where muscle and motor neuron cells can be cultured in separate chambers with their own media reservoirs, but have a bridge connecting them, allowing for axonal sprouting to recapitulate a functional NMJ. Santhanam et al. (2018) developed a microfluidic in vitro NMJ model for drug toxicity testing where human skeletal myoblasts were co-cultured with human motoneurons (MNs) in separate PDMS chambers with a microtunnel array connecting the two chambers as described, allowing for axonal outgrowth. The MNs were electrically stimulated via electrodes, with the resulting myotube contractions exhibiting dose-dependent responses to the toxins—Bungarotoxin, BOTOX^®^, and curare (Santhanam et al. 2018). Osaki et al. utilized a similar setup to develop the first ALS-on-a-chip model by co-culturing iPSC-derived skeletal muscle cells connected via a collagen gel bridge to ALS patient-derived MN spheroids that were opto-genetically engineered to allow for optically stimulated muscle contraction with optical stimulation (Fig. 2C) (Osaki et al. 2018) Their model, measured via micropillar displacement, exhibited ALS pathological features with reduced muscle contraction force (Fig. 2D), that improved upon treatment with ALS drug candidates rapamycin and bosutinib, thus creating a platform for ALS drug screening and disease modeling. Another ALS-on-a-chip platform with iPSC-derived MNs from ALS patients was used by Guo et al. (2020) to investigate NMJ functionality in ALS lines with mutated genes (Guo et al. 2020). Using the same opto-genetic system as Osaki et al. Vila et al. (2019) developed an in vitro microfluidic model to characterize impaired NMJ in MG by co-culturing human skeletal myoblasts and optogenetically engineered neurospheres derived from the same donor, creating the first patient-specific human NMJ (hNMJ). The group recapitulated diseased NMJ pathology by subjecting their NMJ model to serum derived from MG patients and observing diminished NMJ function, as skeletal muscle cells failed to contract upon optical stimulation of MNs. Building upon this platform, the same group incorporated automated video-processing algorithms to create a diagnostic tool that automatically quantifies NMJ function and MG severity in a high-throughput manner based on sera from MG donors (Vila et al. 2021). Another opto-genetically engineered NMJ model was recently developed by Solomon et al. to test the functionality of NMJ in response to vecuronium, a competitive inhibitor of acetylcholine receptors (AChR) (Solomon et al. 2021). The first mature hNMJ model was observed by Bakooshli where primary human myoblasts and human pluripotent stem cell (hPSC)-derived MNs were directed to self-assemble in 3D co-culture, modeling NMJ function in healthy versus MG-afflicted conditions. Mature functional NMJs were observed generating calcium transients in response to glutamate stimuli with and without MG patient-derived IgG treatments. Brack et al.’s (2019) model was the first in vitro hNMJ model to demonstrate the upregulated expression of adult AChR epsilon in muscle fibers, thus creating a mature hNMJ platform to study pathology and development of diseases that affect adult NMJs. A recent study led by Faustino Martins et al. created a breakthrough in generating self-assembled 3D human neuromuscular organoids (NMOs) containing supporting terminal Schwann cells and functional NMJs capable of modeling MG pathology through IgG treatment from MG patients. The 3D NMOs were developed from hiPSC-derived neuro-mesodermal progenitors and were capable of being used for long-term culture studies, with the NMJs reported to being stable up to 150 days (Faustino Martins et al. 2020).

In the context of personalized disease modeling, Maffioletti et al. developed an in vitro humanized muscular dystrophy model by generating iPSC-differentiated skeletal muscles derived from patients with DMD, limb-girdle muscular dystrophy, and skeletal laminopathies. Using this 3D platform, abnormal phenotypes of muscular dystrophies were observed, thus providing a platform to study the development of various muscular disorders and potential treatment regimen (Maffioletti et al. 2018).

Recently, Mondrinos et al. (2021) developed an in vitro muscle model where MSC-derived muscle cells were allowed to “sculpt” themselves into muscle tissue via anisotropic contraction post differentiation with functionalized nodes in the PDMS mold serving as “muscle anchors” to prevent detachment. This proof-of-concept model was used to mimic oxidative injury through hydrogen peroxide treatment, and the therapeutic effect of the drugOlaparib and a combination of retinoic acid (RA) and omega-3 fatty acid eicosapentaenoic acid (EPA) pre-treatments was observed to rescue citrate synthase (CS) activity. By adding a tumor compartment containing A549 spheroids, lung fibroblasts, and macrophages into a separate microfluidic chamber, a model of lung cancer cachexia was developed based on this aforementioned oxidative injury model (Mondrinos et al. 2021).

The utilization of 3D nozzle-based bioprinting technology has also been very recently applied to the fabrication of NMJ to recapitulate the complex 3D NMJ structure more closely by spatial cell patterning (Kong et al. 2021; Sanz et al. 2021); however, 3D in vitro NMJ models generated from microfluidic devices still remain a more widely used alternative to accommodate for the different media requirements of muscle and neuron cells.

The applications of 3D printing technology coupled with micro-fluidics to generate 3D in vitro muscle models, as demonstrated by previously mentioned examples, possess significant potential for the future of muscle development studies, disease modeling, and drug screening compared to 2D culture models. However, the ability to create a reproducible, high throughput in vitro muscle model for disease modeling and drug screening remains a huge challenge.

## 3D printed in vitro model in toxicology: challenge and perspectives

**Fig. 3:**
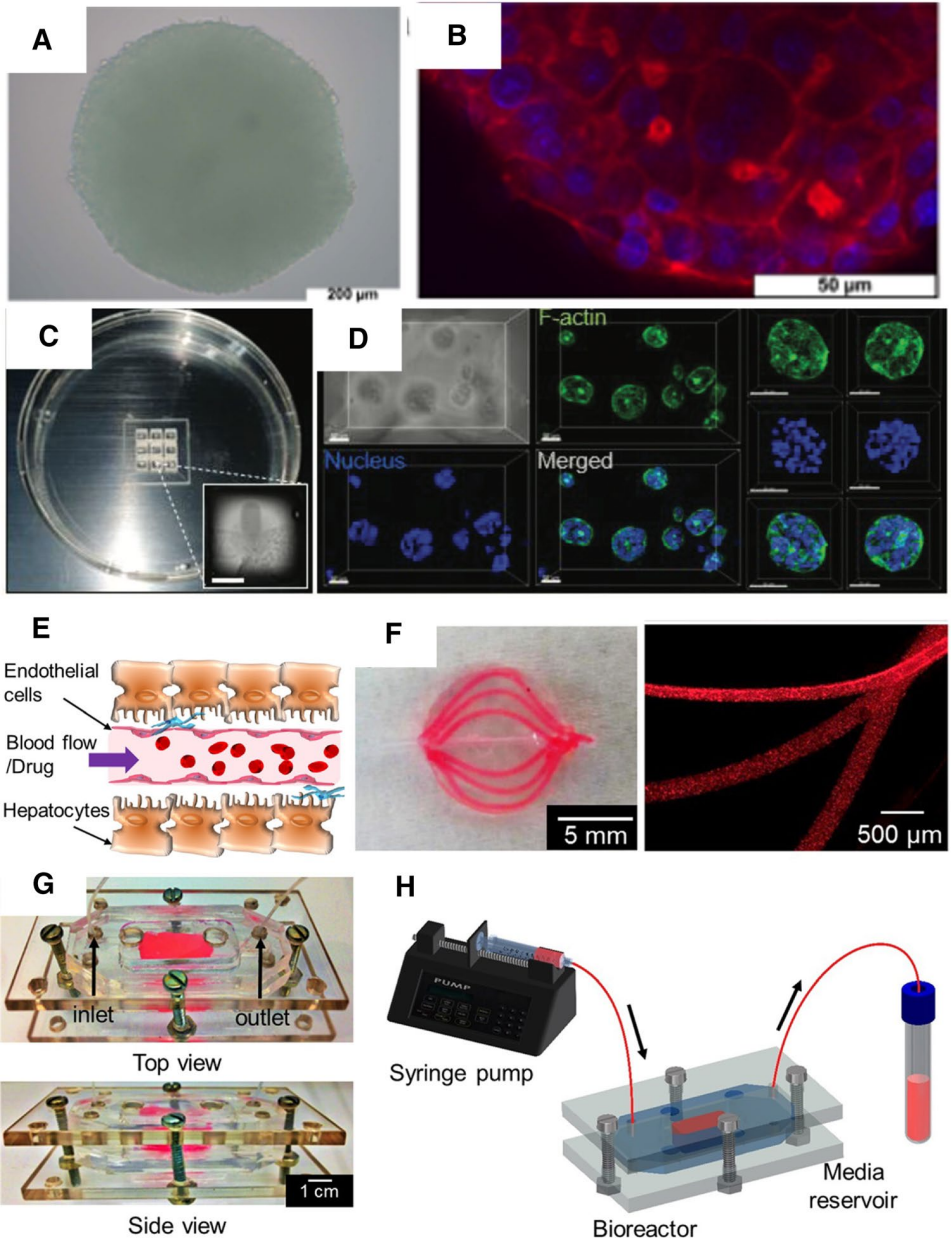
Evolution of in vitro model of complex tissue. **A**, **B** Early liver spheroid culture (Mueller et al. 2014); **C**, **D** liver spheroid culture for in situ quantification (Hong and Song 2021); **E**–**H** 3D printed vascularized liver model (Massa et al. 2017)

With the development of both 3D culture and bio-fabrication technologies, 3D in vitro tissue models have been recognized as an advanced approach for toxicity studies as they provide controllable variations to identify mechanisms of treatment response; close imitation of the microenvironment and physiology of the native tissue; and high efficiency and throughput of model establishment. In an early study, HepaRG and HepG2 spheroids (Fig. 3A, B) were fabricated with a hanging drop system and tested for toxicity response against aflatoxin B1, amiodarone, valproic and chlorpromazine. Compared to the 2D culture, the spheroids showed increased metabolic activities as well as increased gluconeogenesis, cell polarization, and diffusion barrier effect of ECM with the drugs of interest. These distinctions in the drug response between 2D and 3D models demonstrate the importance of the in vivo environment in evaluating toxicity (Mueller et al. 2014). Parallelly, Ramaiahgari et al. (2014) used Matrigel to 3D culture HepG2 cells. The size of the spheroids was regulated by the bottom surface area of the well plate and the amounts of cells. Increased cell polarity, functionality and thereby the sensitivity to the hepatotoxic drugs were also observed.

For better control over the remodeling and architecture recapture of the complex tissue, 3D printing has been involved in the production of the in vitro models, resulting in an improved mimicry of the biochemical, histological and functional recapitulation, as well as addressing the different study purposes. For example, micro-extrusion-based printing was employed to optimize a 3D liver in vitro model. Alginate, gelatin and Matrigel were blended at an optimized ratio for printability, cell viability, metabolism and long-term stability of the scaffold. The viability and secretion of albumin were maintained up to 21 days, indicating the potential of the model for chronic toxicity testing (Schmidt et al. 2020). On the other hand, the rapid yet well-controlled production of the liver spheroid by 3D printing enabled the quantitative evaluation of drug-induced toxicity in an in situ manner (Fig. 3C, D), which can play an accelerating role in the long and expensive drug development process (Hong and Song 2021). One of the important complexed features of the native tissues, which should be taken into consideration for toxicity assessment, is that they are highly vascularized (Fig. 3E). Utilizing extrusion-based 3D printing, Massa et al. fabricated 3D vascularized liver model with perfusable channels (Fig. 3F–H) involving an endothelial layer that barriers the diffusion of molecules. The model was tested for the toxicity study of acetaminophen, which has been revealed to damage the endothelial cells in the liver sinusoid. In this model, the toxicity effect of the drug was tested on the endothelial layer and the HepG2/C3A cell protected by the endothelial layer, better mimicking the in vivo exposure scenario (Massa et al. 2017).

While 3D bio-printing offers many advantages, there are still challenges and limitations to overcome, as well as room to improve.

To successfully replicate complicated tissue in vitro, a model should not only reproduce its histological and biochemical characteristics, but also its functions, such as metabolite exchange, nutrient transportation and contraction. Ahn et al., for example, constructed a 3D heart cantilever based on rat cardiomyocytes, in which a piece of cardiac tissue was remodeled, and the contraction function was recapitulated and evaluated. Dosed with titanium dioxide at 100 μg/mL, they observed a gradual decrease in the contractile force, and the spontaneous beating ceased in 24 h (Ahn et al. 2018). The result did not align with a previous study with a 2D culture model, where the spontaneous beating of the rat cardiomyocytes was maintained despite the reduced beating rate and amplitude (Jawad et al. 2011). There are a few hypotheses as to why the 3D heart tissue stopped beating, including: (1) cardiomyocytes are more resistant to the titanium dioxide toxicity in 2D than in 3D culture; (2) the remaining contractility of the cardiomyocytes after dosing was enough to be observed on the single cell level, but not enough to support the beating function; and (3) the toxicity toward the tissue ECM was reflected in the 3D model but neglected in 2D culture. In any case, the difference between the two studies emphasizes the importance of function recapitulation in the in vitro modeling. However, the gap between the exquisite native tissue and our limited understanding of the *in vivo* conditions and appropriate fabrication technology remains a key difficulty and hot topic in ongoing research.

### Tissue complexity

The complexity of the tissue is one of the challenges in reproducing a native tissue. Regardless of the biomaterial sources available, it remains a challenge to fully represent the biophysical and biochemical features of the native ECM in an artificial bio-microenvironment. One solution for the dilemma is to incorporate dECM in the biomaterial applied in the 3D printing. As reported by Ma et al. (2018a, b), the photo-patterning of cells and stiffness of the scaffold were precisely regulated via 3D printing of the dECM-blended bioink to provide biomimetic physical signals to the cells, while the biochemical cues embedded in the native liver ECM were simultaneously delivered. Similar results were achieved in a later study by another group (Mao et al. 2020). For control of the biochemical cues individually in the scaffold, highly engineered synthetic material customized with biomolecules of interest has been developed to facilitate the establishment of 3D culture systems. In an early collective study conducted by Taubenberger et al., PEG was decorated with biochemical mediators, such as metalloproteinase-cleavage spot, ECM mimicking cell adhesion peptides and a set of growth factors. The highly programmable platform they developed was able to create an in vitro bio-microenvironment with multiplexed biochemical cues and controlled mechanical properties of the matrix (Taubenberger et al. 2016). Another challenge is that the ECM is highly dynamic. Material properties must be carefully tuned to match the progression of the in vitro model and physiological process. For example, in the cardiac micro-tissue fabricated via DLP printing, the crosslinking density of the scaffold made of GelMA was carefully tuned, so that the degradation rate of the scaffold matched the ECM deposition of human cardiac fibroblasts (HCF), which essentially supported the maturation and contraction phenotype of the artificial tissue over 7 days (Miller et al. 2021). To improve the mimicry, a dynamic culture system could be combined with the artificial tissue. For example, a microfluidic chip with pressure channels was designed for colon tumor organoid culture to mimic the peristalsis, which is an important feature of their native microenvironment. The proliferation and organoid size were significantly increased compared to static culture as the media flowed through the pressure channels and mechanical stimulus was delivered. The peristalsis-stimulated organoid also showed decreased uptake and response to ellipticine-laden micelle dosing, implying that genuine recapitulation of ECM dynamics may have a significant impact on the in vitro model's drug/toxin response (Fang et al. 2021).

On the other hand, native tissue has a complex cell composition, whereas much of the earlier research used only a few cell lines. It is an effective approach to simplify the model for certain scientific problems, but it hinders the recapitulation of tissue integrity, functionality, and response to drug/toxin doses. Organoid and in situ differentiation offer significant advantages in breaking down this barrier. Gu et al. created 3D neuron constructs from human neuron stem cells using extrusion-based 3D printing. After in situ differentiation of the stem cells, a neuron micro-tissue composed of functional neurons and supporting neuroglia was obtained (Gu et al. 2016). A follow-up study using iPSCs was conducted by the same group, demonstrating the strategy's potential for application in a variety of tissues (Gu et al. 2017). This method is particularly useful for models that require difficult-to-obtain cell sources. For example, because of the non-proliferating nature of cardiomyocytes and the requirement for high cardiomyocyte density, it has been difficult to establish an in vitro cardiac model in a large dimension and with integrated anatomy structures. Kupfer et al. (2020) used in situ differentiation of 3D printing iPSCs to create a cardiac organoid with highly biomimetic chamber structure and pumping activities on a 1.3 cm scale. While the organoid technology and in situ differentiation provide a versatile strategy to achieve multicellular construct, it is difficult to control the ratio of the cellular component. For designated cell composition, 3D printing could also be exploited as it provides high resolution for cell deposition. Focusing on the subject has resulted in significant progress. Because of their high yet biocompatible printing resolution, light-assisted printing processes, such as stereolithography and DLP printing, are intrinsically advantageous for multicellular printing. Furthermore, multiplexed printing systems have been developed to aid the facile multicellular printing; for example, multi-head and core–shell structured nozzles for extrusion and inkjet-based bioprinting have been developed. In place of the distinct flow mechanics at the micro-scale, microfluidic-based strategies can also be incorporated to the conventional 3D printing processes for precise control on the heterogeneity of the bioink. Ashammakhi et al. (2019) have provided a more in-depth review on the topic.

### Systematic recapitulation

Another gap to be filled is the lack of mimicry on the systematic level. Many pharmacological and toxicological studies are hampered by poor in vitro–in vivo correlation because the response is usually generated by organ-organ interaction rather than a single organ or tissue. To address this concern, multi-organ-on-a-chip models incorporating multiple tissue models linked to a shared medium circulation system have been developed. Microfluidic devices have emerged as a powerful platform with individual chambers for the culture of each tissue and controlled flow of media enabled by designated channels, and extensive work has been devoted to such culture systems, resulting in improved drug response prediction outcomes (Sung 2021). Further improvement could be achieved by incorporating the evolving in vitro tissue models as discussed above as well as more customized and tailored design in the microfluidic devices for better mimicry of the flow dynamics and substance exchange (Goldstein et al. 2021).

In addition, it is necessary for systematic modeling to involve the in vitro recapitulation of the immune system, which plays a critical role in many disease progression and pharmacological/toxicological responses, and has become increasingly important in the development of therapeutics for many conditions. Keeping this in mind, recent studies have focused on incorporating immune components into in vitro models and have achieved effective results. iPSC and THP-1-derived macrophages, for example, were incorporated into the in vitro glioblastoma microenvironment model to mimic the infiltration of macrophage alongside microglia via DLP printing. The engagement of the macrophages resulted in a significantly more authentic recapitulation of the original tissue, which had a substantial impact on the drug response of the model (Tang et al. 2020). THP-1 was also used to investigate the general pro-inflammatory response in a human-based multi-organ-on-a-chip model which involved hepatocyte, cardiomyocyte and skeletal muscle myoblasts (Sasserath et al. 2020). However, most of the work has been devoted to the innate immune system, while the in vitro modeling of the adaptive immune system remains to be explored. In fact, the state-of-the-art in vitro modeling of the immune system is still in its early stages. A more in-depth and comprehensive review regarding the topic has been provided by Polini et al. (2019).

### Microarchitecture

**Fig. 4:**
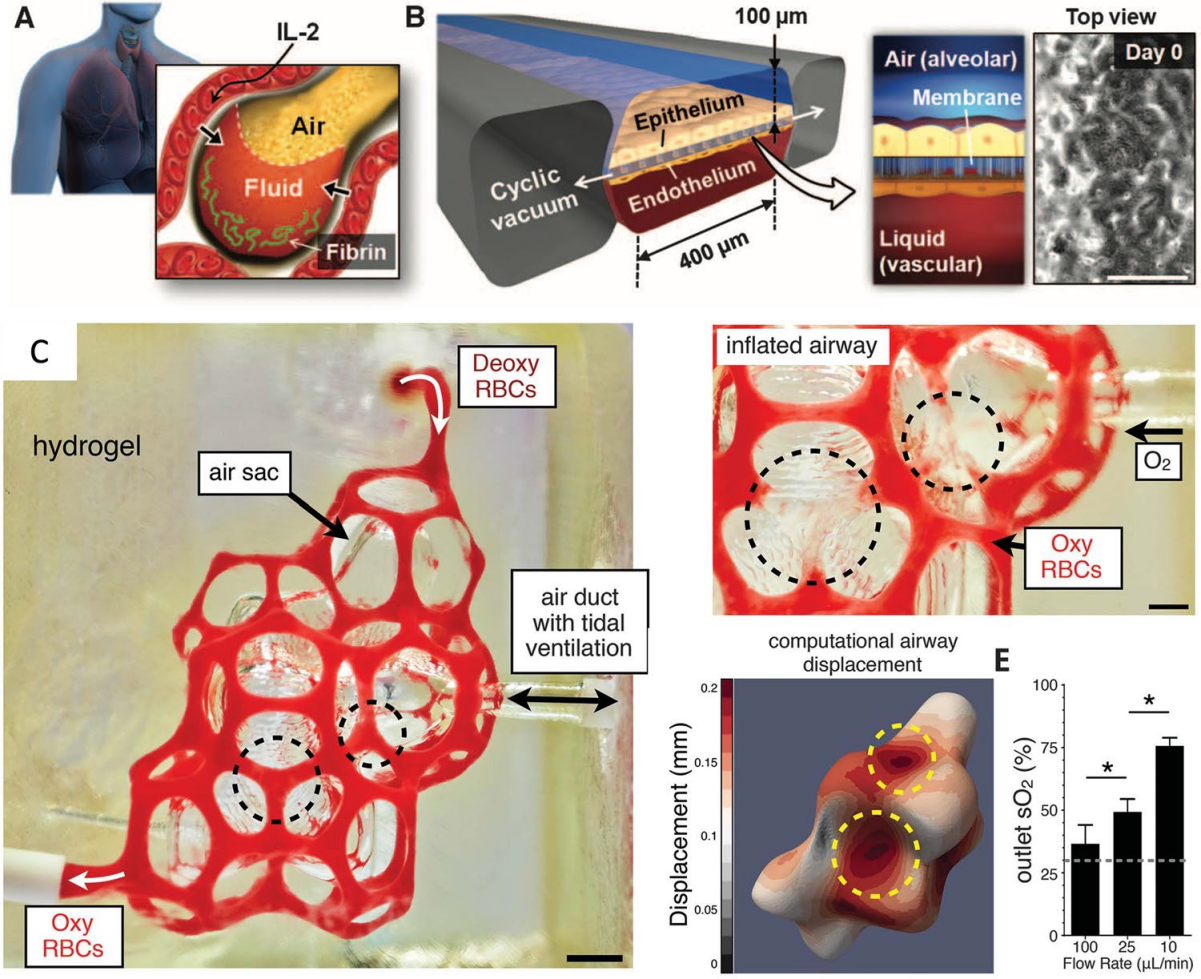
**A** hierarchical and entangled structure of human lung alveolus (Huh et al. 2012); **B** the air–liquid interface model based on a PDMS chamber (Huh et al. 2012); **C** genuine recapitulation of the complexed alveolus microstructure by photo-absorbent-assisted stereolithography (Grigoryan et al. 2019)

Furthermore, due to the limited resolution of bioprinting and cell deposition, it is difficult to recreate the microarchitecture and hierarchical nature of the tissue, which is particularly important in the integrated functionality of certain organs, such as the lungs, kidneys, and livers. The alveoli in the lungs, for example, are physically air sacs held and entangled by a network of capillaries (Fig. 4A). This extremely sophisticated structure is necessary for the exchange of oxygen and carbon dioxide between the pulmonary and circulatory systems, but it also adds a significant challenge to the fabrication process, particularly with soft materials. Earlier studies focused on multilayer 3D culture integrated on a PDMS chamber to capture the air–liquid interface in the alveolus for disease modeling and drug testing (Fig. 4B) (Benam et al. 2016; Huh et al. 2012). While the biochemical and histological characteristics of the tissue were successfully replicated to some extent, the membrane thickness of the blood vessels and the cell-liquid ratio were not fully recapitulated, resulting in inaccurate capture of tissue metabolism and biochemical signaling (Shrestha et al. 2020). The advanced 3D printing technique can improve the recapitulation of the alveolar microarchitecture. Innovatively applying food dye as a photo-absorber, Grigoryan et al. enhanced the printing resolution of soft hydrogel, PEGDA (*M*_w_ = 6 kDa), in DLP-based 3D printing  (Fig. 4C). This allowed the printing of the complexed microstructure of alveolar with a biocompatible bioink, in which oxygenation and flow of human red blood cells was supported and vascularization of human umbilical vein endothelial cells (HUVECs) was achieved (Grigoryan et al. 2019). One limitation of this study is that the native alveolar cell composition was not included or tested, which limits the model's application in drug and toxin studies. Nonetheless, it is stated that the recapitulation of the exquisite native tissue's microarchitecture is within reach owing to the rising resolution of 3D printing techniques and a wider range of biomaterials.

### Vascularization

As the nature route of nutrient and oxygen transportation and an essential part in the induction of organogenesis (Ding et al. 2010), vasculatures remain a stumbling block in both the maturation of organoids (Vargas-Valderrama et al. 2020) and the 3D fabrication of larger in vitro tissues. Currently, the major approach to introduce the vascularization to an in vitro model is to involve a sacrificial template. For example, Skylar-Scott et al. proposed a sacrificial writing into functional tissue (SWIFT) method, in which they compact a large volume of organoids in a thermal-gelling ECM and directly write the vascular channels with gelatin ink in an embedding manner at 0–4 °C. After printing, the gelatin was removed by perfusion as the construction was warmed up to 37 °C for future culture. This novel strategy enabled addition of vasculature and large-dimension culture of organoids (Skylar-Scott et al. 2019). The sacrificial gelatin ink could also facilitate direct printing of vasculatures in a co-axial extrusion-based 3D printing set-up. As reported by Shao et al. perfusable core–shell constructs with a scale larger than 1 cm have been printed with HUVECs mixed in the inner gelatin phase and tissue cells (human breast cancer cell MDA-MB-231 and mouse osteoblast MC3T3-E1) laden in the outer GelMA phase. After removal of gelatin and auto-seeding of HUVECs, the construct remodeled into a vasculature and was cultured for over 20 days (Shao et al. 2020). The phase-transition removal of gelatin is more biocompatible to avoid unwanted chemical stimulation compared to alginate-Ca and Pluronic, which are commonly used in earlier work, but the temperature shock involved in this process should be taken into consideration for specific cells and models (Lindquist and Mertens 2018). Moreover, the process to remove the template still hinders the efficient fabrication of the in vitro model. In contrast, DLP printing remains advantageous to fabricate vasculatures directly and rapidly. Zhu et al. reported the DLP-based continuous bioprinting of a pre-vascularized in vitro tissue with gradient channel diameter and biomimetic branching configuration. Facilitated with a motorized syringe pump, each construct was printed within 1 min; lumen-like structure and tight junctions of the endothelium was developed after culture for 1 week (Zhu et al. 2017). Despite the evolving techniques to produce vascularized perfusable channels, most work was conducted with primary HUVECs, which are isolated from an identical organ and stage of human life (Cao et al. 2017). However, the endothelium is highly heterogeneous throughout the different organs in terms of histological features, metabolism, angiogenesis and involvement in the immune response (Przysinda et al. 2020). It is noteworthy that in the development of an organ-specific vasculature, the diverse phenotypes of the endothelium should be recognized and well characterized.

### High throughput

High-throughput screening (HTS) systems are widely used by the pharmaceutical industry as an efficient method to process large numbers of compounds and molecular targets in a rapid manner. As such, pharmaceutical companies are turning to the use of 3D biomimetic human tissues in HTS format for preclinical toxicity testing of potential drug candidates. 3D bioprinting approaches, such as inkjet and micro-extrusion-based processes, are limited in scalability, resolution, patterning flexibility, and/or speed, which makes them unsuitable for high-throughput fabrication of complex cell-laden 3D microstructures within multi-well plates commonly used in HTS systems for drug screening and assay development. These shortcomings severely limit the widespread adoption of 3D printed cell culture platforms since researchers often rely on products configured to interface with commonly used lab instruments and equipment. Recently, DLP printing has made HTS possible. With rational integration of the micro-continuous projection printing and the automated well plate registration, Hwang et al. managed to fabricate complex 3D bio-constructs directly in a well plate rapidly and consistently. Depending on the complexity of the tissue construct, the total fabrication time for a fully populated 96-well plate typically ranges from 20 to 40 min (Hwang et al. 2021). Future work for printing in a 384-well plate or more wells is yet to be developed for HTS applications.

### Concluding remarks

3D bioprinting has made significant progress in in vitro recapitulation of complex tissues. By applying an appropriate printing technique and biomaterial, accurate recapture of the ECM composition, cell population, bio-microenvironment, organ microarchitecture, and tissue functionality has been achieved in a variety of tissues, such as cancer, heart, muscle, liver, kidney, and will continue to be broadened and enhanced as the studies go on. Despite the encouraging results being achieved, it is noteworthy that they are usually labor and technique intensive. To bridge the gap between fundamental research and pragmatic applications, the scalability and reproducibility of the modeling strategies must also be addressed. In addition, with the arising need in the evaluation of therapeutic and substance in subdivided population and in the point-of-care scenario, the required time of production of personalized in vitro model must also be improved.
